# Lymphangiome vulvaire acquis après traitement d'un cancer gynécologique

**DOI:** 10.11604/pamj.2015.20.126.6108

**Published:** 2015-02-13

**Authors:** Boufettal Houssine, Samouh Naïma

**Affiliations:** 1 Obstetrics and Gynecology Department, Ibn Rochd University Hospital, Hassan 2 University, Faculty of Medicine and Pharmacy, Casablanca, Morocco

**Keywords:** Lymphangiome, vulve, acquis, Lymphangioma, vulva, acquired

## Image en medicine

Une patiente âgée de 38 ans, mère de deux enfants. Elle était opérée il y'a dix années pour un carcinome épidermoïde du col utérin, stade IIb proximal, par une colpectomie élargie avec lymphadénectomie pelvienne après une association radiothérapie et chimiothérapie première. Les suites opératoires étaient simples. Aucune récidive n’était notée. Elle présentait dans les suites opératoires tardives, un lymphœdème des membres inférieures, pour lequel elle bénéficiait de plusieurs séances de kinésithérapie, avec bonne évolution. Depuis deux années, elle présentait des lésions vésiculeuses des grandes lèvres, qui s'xacerbaient après le rasage (figure). Une biopsie cutanée des lésions vulvaires montrait un lymphangiome cutané superficiel et circonscrit.

**Figure 1:**
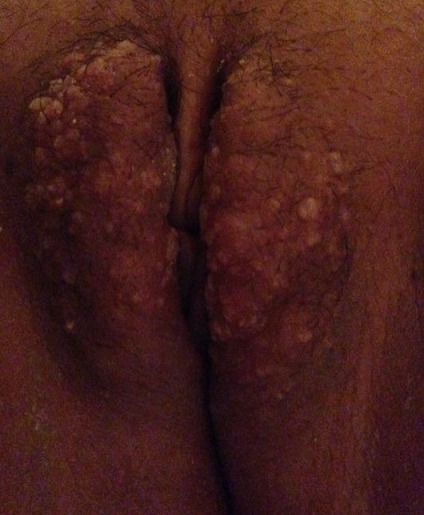
Lymphangiome vulvaire secondaire à un traitement d'un cancer gynécologique, avec des lésions vésiculeuses des grandes lèvres

